# Molecular mechanism of the type 2 defense-associated reverse transcriptase

**DOI:** 10.1093/nar/gkaf1135

**Published:** 2025-11-08

**Authors:** Zhikun Liu, Fumeng Liao, Wenqi Wu, Chendi Zhang, Caidie Yue, Aoyan Chen, Shuqin Zhang, Yingcan Liu, Bin Liu, Jie Han, Chuyun Zhang, Xiaoshen Wang, Xuzichao Li, Zhuang Li, Heng Zhang, Hang Yin

**Affiliations:** Tianjin Medical University Cancer Institute and Hospital, State Key Laboratory of Experimental Hematology, Key Laboratory of Immune Microenvironment and Disease (Ministry of Education), The Province and Ministry Co-sponsored Collaborative Innovation Center for Medical Epigenetics, National Clinical Research Center for Cancer, Tianjin Institute of Immunology, School of Basic Medical Sciences, Tianjin Medical University, Tianjin 300070, China; Tianjin Key Laboratory of Cellular Homeostasis and Disease, Tianjin’s Clinical Research Center for Cancer, Key Laboratory of Cancer Prevention and Therapy, Department of Biochemistry and Molecular Biology, Tianjin Medical University, Tianjin 300070, China; Tianjin Medical University Cancer Institute and Hospital, State Key Laboratory of Experimental Hematology, Key Laboratory of Immune Microenvironment and Disease (Ministry of Education), The Province and Ministry Co-sponsored Collaborative Innovation Center for Medical Epigenetics, National Clinical Research Center for Cancer, Tianjin Institute of Immunology, School of Basic Medical Sciences, Tianjin Medical University, Tianjin 300070, China; Tianjin Key Laboratory of Cellular Homeostasis and Disease, Tianjin’s Clinical Research Center for Cancer, Key Laboratory of Cancer Prevention and Therapy, Department of Biochemistry and Molecular Biology, Tianjin Medical University, Tianjin 300070, China; Tianjin Medical University Cancer Institute and Hospital, State Key Laboratory of Experimental Hematology, Key Laboratory of Immune Microenvironment and Disease (Ministry of Education), The Province and Ministry Co-sponsored Collaborative Innovation Center for Medical Epigenetics, National Clinical Research Center for Cancer, Tianjin Institute of Immunology, School of Basic Medical Sciences, Tianjin Medical University, Tianjin 300070, China; Department of Gastric Surgery, Tianjin Medical University Cancer Institute and Hospital, National Clinical Research Center for Cancer, Key Laboratory of Cancer Prevention and Therapy, Tianjin’s Clinical Research Center for Cancer, Tianjin 300060, China; State Key Laboratory of Biocatalysis and Enzyme Engineering, School of Life Sciences, Hubei University, Wuhan 430062, China; Tianjin Medical University Cancer Institute and Hospital, State Key Laboratory of Experimental Hematology, Key Laboratory of Immune Microenvironment and Disease (Ministry of Education), The Province and Ministry Co-sponsored Collaborative Innovation Center for Medical Epigenetics, National Clinical Research Center for Cancer, Tianjin Institute of Immunology, School of Basic Medical Sciences, Tianjin Medical University, Tianjin 300070, China; Tianjin Medical University Cancer Institute and Hospital, State Key Laboratory of Experimental Hematology, Key Laboratory of Immune Microenvironment and Disease (Ministry of Education), The Province and Ministry Co-sponsored Collaborative Innovation Center for Medical Epigenetics, National Clinical Research Center for Cancer, Tianjin Institute of Immunology, School of Basic Medical Sciences, Tianjin Medical University, Tianjin 300070, China; Tianjin Medical University Cancer Institute and Hospital, State Key Laboratory of Experimental Hematology, Key Laboratory of Immune Microenvironment and Disease (Ministry of Education), The Province and Ministry Co-sponsored Collaborative Innovation Center for Medical Epigenetics, National Clinical Research Center for Cancer, Tianjin Institute of Immunology, School of Basic Medical Sciences, Tianjin Medical University, Tianjin 300070, China; Tianjin Medical University Cancer Institute and Hospital, State Key Laboratory of Experimental Hematology, Key Laboratory of Immune Microenvironment and Disease (Ministry of Education), The Province and Ministry Co-sponsored Collaborative Innovation Center for Medical Epigenetics, National Clinical Research Center for Cancer, Tianjin Institute of Immunology, School of Basic Medical Sciences, Tianjin Medical University, Tianjin 300070, China; Tianjin Medical University Cancer Institute and Hospital, State Key Laboratory of Experimental Hematology, Key Laboratory of Immune Microenvironment and Disease (Ministry of Education), The Province and Ministry Co-sponsored Collaborative Innovation Center for Medical Epigenetics, National Clinical Research Center for Cancer, Tianjin Institute of Immunology, School of Basic Medical Sciences, Tianjin Medical University, Tianjin 300070, China; Department of Anatomy, School of Basic Medical Sciences, Tianjin Medical University, Tianjin 300070, China; Tianjin Medical University Cancer Institute and Hospital, State Key Laboratory of Experimental Hematology, Key Laboratory of Immune Microenvironment and Disease (Ministry of Education), The Province and Ministry Co-sponsored Collaborative Innovation Center for Medical Epigenetics, National Clinical Research Center for Cancer, Tianjin Institute of Immunology, School of Basic Medical Sciences, Tianjin Medical University, Tianjin 300070, China; Tianjin Medical University Cancer Institute and Hospital, State Key Laboratory of Experimental Hematology, Key Laboratory of Immune Microenvironment and Disease (Ministry of Education), The Province and Ministry Co-sponsored Collaborative Innovation Center for Medical Epigenetics, National Clinical Research Center for Cancer, Tianjin Institute of Immunology, School of Basic Medical Sciences, Tianjin Medical University, Tianjin 300070, China; Tianjin Medical University Cancer Institute and Hospital, State Key Laboratory of Experimental Hematology, Key Laboratory of Immune Microenvironment and Disease (Ministry of Education), The Province and Ministry Co-sponsored Collaborative Innovation Center for Medical Epigenetics, National Clinical Research Center for Cancer, Tianjin Institute of Immunology, School of Basic Medical Sciences, Tianjin Medical University, Tianjin 300070, China; State Key Laboratory of Biocatalysis and Enzyme Engineering, School of Life Sciences, Hubei University, Wuhan 430062, China; Tianjin Medical University Cancer Institute and Hospital, State Key Laboratory of Experimental Hematology, Key Laboratory of Immune Microenvironment and Disease (Ministry of Education), The Province and Ministry Co-sponsored Collaborative Innovation Center for Medical Epigenetics, National Clinical Research Center for Cancer, Tianjin Institute of Immunology, School of Basic Medical Sciences, Tianjin Medical University, Tianjin 300070, China; Tianjin Key Laboratory of Cellular Homeostasis and Disease, Tianjin’s Clinical Research Center for Cancer, Key Laboratory of Cancer Prevention and Therapy, Department of Biochemistry and Molecular Biology, Tianjin Medical University, Tianjin 300070, China; Tianjin Medical University Cancer Institute and Hospital, State Key Laboratory of Experimental Hematology, Key Laboratory of Immune Microenvironment and Disease (Ministry of Education), The Province and Ministry Co-sponsored Collaborative Innovation Center for Medical Epigenetics, National Clinical Research Center for Cancer, Tianjin Institute of Immunology, School of Basic Medical Sciences, Tianjin Medical University, Tianjin 300070, China; Department of Pharmacology, School of Basic Medical Sciences, Tianjin Medical University, Tianjin 300070, China

## Abstract

Defense-associated reverse transcriptase (DRT) systems play a crucial role in prokaryotic defense mechanisms against phage infections. Among the DRT family, DRT2, DRT3, and DRT9 systems employ protein–noncoding RNA (ncRNA) co-regulatory mechanisms to execute defense functions. Here, we focus on the DRT2 system from *Klebsiella pneumoniae*, which consists of a reverse transcriptase (RT) and an essential ncRNA component. Using biochemical and structural approaches, we determine the structure of the DRT2 system and reveal detailed interaction modes between the DRT2-RT protein and the ncRNA, especially mediated by specialized anchoring loops and pseudoknot-related structures. The RT protein adopts a conventional “right-hand” fold, while a flexible region of the ncRNA exhibits dynamic conformations, likely serving as the template for reverse transcription. DRT2 mediates reverse transcription through a conserved DDD catalytic triad that coordinates a divalent Mg²⁺ ion. Notably, a short DNA primer-ncRNA duplex is accommodated in a positively charged pocket formed by the thumb and fingers domains, and both interaction analysis and mutagenesis studies confirm that duplex stabilization is essential for activity. Structural comparison and phylogenetic studies of DRT2 and other RT proteins, such as group II introns and UG/Abi RTs, highlight the unique adaptation with a straight extended thumb domain and specialized structures for ncRNA-binding, exemplifying an evolutionary trajectory of RT proteins. In conclusion, our findings expand the understanding of the distinctive characteristics of the DRT2 system and the diversity of prokaryotic antiviral strategies.

## Graphical Abstract

**Graphical Abstract:**
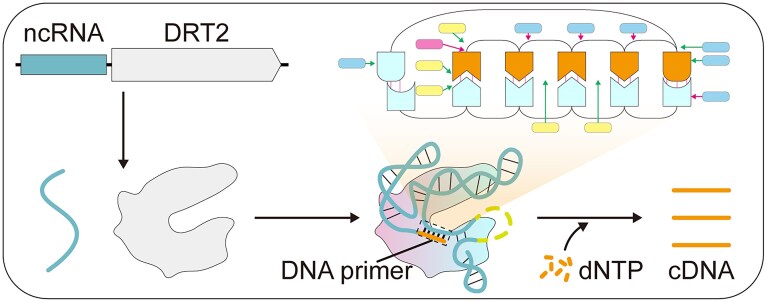

## Introduction

Reverse transcription, the process of RNA-templated DNA synthesis, plays an important role in the transmission of genomic information and the corresponding enzyme responsible for catalyzing this process is known as reverse transcriptase (RT) [1]. Accumulating research on RTs suggests that they tend to be co-opted by cells for specific cellular functions [2]. In eukaryotes, telomerase utilizes RT activity to maintain genome integrity by preserving chromosomal stability [3]. Meanwhile, pre-messenger RNA (mRNA)-processing factor 8 (Prp8), a core component of the eukaryotic spliceosome, induces its activity for pre-mRNA splicing [4]. In contrast, non-long terminal repeat (non-LTR) retrotransposons employ a target-primed reverse transcription (TPRT) mechanism to synthesize complementary DNA (cDNA) directly at chromosomal integration sites nicked by endonucleases [5]. In prokaryotes, RTs are more frequently repurposed for defense-related functions [2, 6]. Most prokaryotic RTs can be phylogenetically classified into several lineages, including group II introns (G2I), diversity-generating retroelements, retrons, Group II-like elements, CRISPR-Cas-associated RTs, abortive infection (Abi) RTs, and unknown groups (UG) [1, 2, 7]. Among UG/Abi RTs, some contain a single RT domain, while others consist of α-helix HEAT-like repeats at the C-terminal, and at least some RTs associated with hydrolase or phosphohydrolase domains for different enzymatic activities [8]. Notably, certain UG/Abi RTs have been experimentally shown to function in defending against phage infection, thus named defense-associated RT (DRT) family.

The DRT family currently includes nine distinct proteins (DRT1-9), each featuring a unique domain composition. DRT1 is associated or fused with C-N hydrolase or phosphohydrolase domains, while DRT7 contains a PrimS domain located at its C-terminal region and DRT8 is characterized by consisting of a PD-(D/E) XK domain. Notably, DRT2, DRT3, and DRT9 contain ncRNA components in addition to their RT domains, and the ncRNAs are experimentally demonstrated to be essential for their defense functions [6, 8]. However, the mechanism by which DRT RT proteins and their ncRNAs collaboratively execute defense functions remains poorly understood. In this study, we focus on the DRT2-RT–ncRNA defense system, employing biochemical and structural approaches to delineate its structural basis and functional mechanisms, expanding our understanding of the diversity of prokaryotic defense strategies.

## Materials and methods

### Protein expression and purification

The *drt2* gene, which encodes the full-length DRT2 protein (GenBank: NC_012731.1), was amplified by polymerase chain reaction and cloned into the pET28-MKH8SUMO vector (Addgene #79526) with an N-terminal His-SUMO fusion tag. The ncRNA sequence was cloned into a pET co-transformation cloning vector (13S-A) (Addgene #48323). Both resultant vectors were then co-transformed into BL21 (DE3) cells for protein expression in lysogeny broth (LB) medium. When the OD600 value reached 0.6, protein expression was induced by adding 0.5 mM isopropyl β-D-l-thiogalactoside (IPTG) at 18°C overnight. The cells were harvested through centrifugation and lysed by sonication in lysis buffer (25 mM Tris-HCl, pH 8.0, 500 mM NaCl, 2 mM β-mercaptoethanol). The supernatant of lysate was loaded into Ni-NTA resin (Qiagen) and the target protein was eluted by elution buffer (25 mM Tris-HCl, pH 8.0, 500 mM NaCl, 2 mM β-mercaptoethanol, 300 mM imidazole) after thorough washing. The His-SUMO tag was then removed using TEV protease. The target protein complex was further purified using HiTrap Q HP column (Cytiva) for ion-exchange chromatography and Superose 6 Increase column (Cytiva) with gel-filtration buffer (25 mM Tris-HCl, pH 8.0, 300 mM NaCl, 2 mM DTT). The peak fractions containing target protein were collected and further analyzed with sodium dodecyl sulphate-polyacrylamide gel electrophoresis (SDS-PAGE) and urea-polyacrylamide gel electrophoresis (PAGE). DRT2 mutants carrying point mutations were generated using the QuickChange mutagenesis kit (Takara) and purified using the same procedure.

### Cryo-EM sample preparation and data collection

For cryo-Electron Microscopy (cryo-EM) sample preparation, an aliquot of 4 μl DRT2-RT-ncRNA binary complex was applied to glow-discharged Quantifoil holey carbon grid (Au, R1.2/1.3, 300 mesh). The grid was blotted with force 3 for 6 s at 4°C and 100% humidity and plunge-frozen into liquid ethane which was cooled by liquid nitrogen using Vitrobot Mark IV (FEI Thermo Fisher). Cryo-EM data were collected with a Titan Krios microscope (FEI) operated at 300 kV, and images were collected using EPU [9] at a nominal magnification of 105 000× (resulting in a calibrated physical pixel size of 0.85 Å/pixel) with a defocus range from -1.2 to -2.2 μm. The images were recorded on a K3 summit electron direct detector in super-resolution mode at the end of a GIF-Quantum energy filter operated with a slit width of 20 eV. A dose rate of 15 electrons per pixel per second and an exposure time of 2.5 s were used, generating 40 movie frames with a total dose of ∼54 electrons/Å^2^. Finally, a total of 4009 movie stacks were collected.

### Cryo-EM image processing

The movie frames were imported into RELION-3 [10], aligned using MotionCor2 [11] with a bin factor of 2, and subjected to on-the-fly contrast transfer function estimation using Gctf [12]. A total of 2153 585 particles were picked using template-based picking and extracted from the dose-weighted micrographs. Following 2D classification, 401 138 particles were selected for further ab-initio reconstruction. After several rounds of heterogeneous refinement, the best volume features well-defined secondary structures in cryoSPARC [13]. Ultimately, 347 211 particles were used for final 3D refinement, achieving a resolution of 3.49 Å.

### Model building and refinement

The initial atomic model of DRT2 was generated using Alphafold3 [14], provided with the sequences of DRT2 and ncRNA. Then, the protein model was fit into the cryo-EM density map using ChimeraX [15] and the ncRNA was manually refined to trace the density map and map to the sequence by Coot [16]. Further iterative refinements were performed by *phenix.real_space_refine* [17] to generate the final model. The quality of the final model was validated by MolProbity in Phenix [18]. Data collection and model refinement statistics are concluded in Table S1.

### *In vitro* reverse transcription assay

The purified DRT2-RT-ncRNA complex and mutants were diluted to OD260 = 1 in reaction buffer (20 mM Tris-HCl, pH 8.0, 150 mM NaCl, 5 mM MgCl₂, 3 mM DTT, and 5% glycerol). For wild-type DRT2 complex, reactions were initiated by adding increasing concentrations of dNTPs (0-500 μM), carried out at 37°C for 30 min, and terminated by the addition of ethylenediaminetetraacetic acid and proteinase K. For mutants, reactions were performed at 500 μM dNTP under the same conditions. Then the mixture sample was further analyzed by 0.7% agarose gel electrophoresis. The gel was analyzed utilizing an Azure Imaging System.

### Phylogenetic analysis

To analyze the phylogenetic relationships among the homologous structures identified, we collected sequences of the 2138 structures retrieved from the DALI server. These sequences were then clustered using cd-hit (v4.8.1) [19] with a similarity threshold of 0.8, and representative sequences were selected. The redundancy-reduced sequences were aligned using MAFFT (v7.526) [20] with default parameters, followed by trimming of excessive gap regions using trimAl (v1.5) [21]. Phylogenetic tree construction was performed using IQTREE (v2.3.6) [22], with the Modelfinder module [23] selecting LG + R6 as the optimal model. Branch support was evaluated through 1000 iterations of ultraFast bootstrap (UFBoot) resampling [24]. The resulting tree was visualized using the iTOL (v6) platform [25].

### Multiple sequence alignment

To analyze the conservation of the catalytic sites in DRT2, we manually selected several representative homologous structures with high Z-scores from the DALI search results. Their sequences were collected and aligned using MAFFT (v7.526). The aligned sequences were then trimmed using trimAl (v1.5) with default parameters. The final alignment was visualized using T-Coffee (v8.99) [26], and conservation analysis was performed using WebLogo (v2.8.2) [27]. For ncRNA conservation analysis, DRT2 orthologs were selected from the “UG2” systems previously classified [8]. Based on the analysis of evolutionary relationships, several closely related DRT2 systems were identified, and the ncRNA sequences located within 1 kb upstream of the RT genes were retrieved. Multiple sequence alignment of these ncRNAs was performed using the same procedures described above.

## Results

### Overall structure of type 2 defense-associated reverse transcriptase

**Figure 1.**
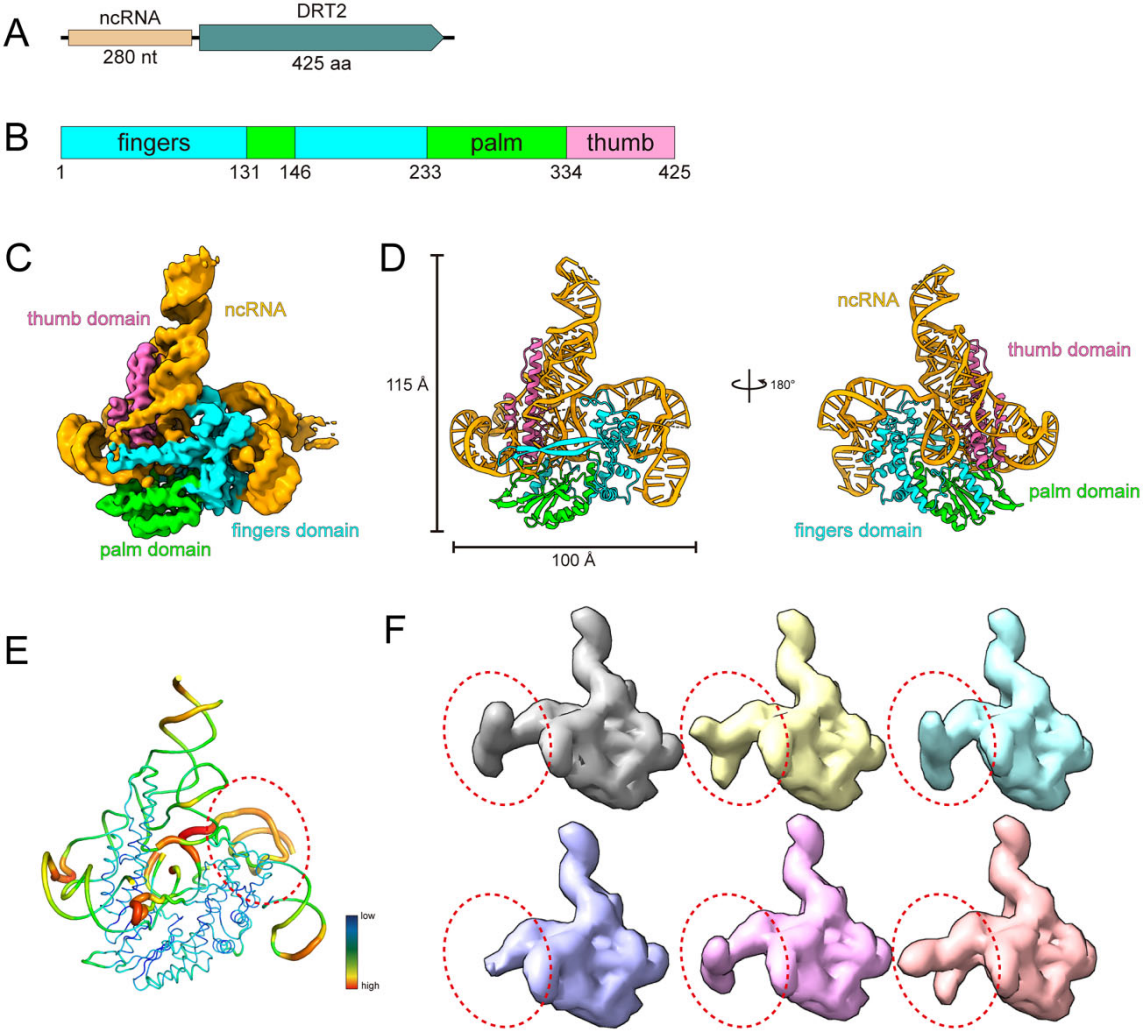
Overall structure of DRT2 system. (**A**) Schematic representation of the DRT2 system from *K. pneumoniae*, and DRT2 system contains a RT and a noncoding RNA (ncRNA), colored in wheat and deepteal, respectively. (**B**) The domain architecture of DRT2 RT protein. The fingers domain is colored in cyan, the palm domain is colored in green and the thumb domain is colored in pink. (**C**) Cryo-EM density map of DRT2 system. The ncRNA is colored in orange and RT domains are colored in the same scheme as in Fig. 1B. (**D**) Atomic model of DRT2 system. The color scheme is the same as Fig. 1B. (**E**) B-factor analysis of DRT2. Model is colored according to B-factors. (**F**) 3D classification of DRT2 system.

The type 2 defense-associated reverse transcriptase (DRT2) has been reported to confer resistance against phage infection [6]. The DRT2 system from *Klebsiella pneumoniae* consists of a 425-amino acid (aa) protein containing only the RT domain and an upstream 280-nucleotide (nt) ncRNA (Fig. 1A). The molecular weight of the DRT2-RT protein is ∼49.7 kDa. DRT2-RT protein comprises three domains, including a fingers domain (residues 1–131 and 146–233), a palm domain (residues 132–145 and 234–334), and a thumb domain (residues 335–425) (Fig. 1B). To elucidate the molecular basis of DRT2-mediated defense function, we expressed and purified the DRT2 system, and the peak containing target complex eluted at 16–17 ml from Superose 6 Increase column. The peak was further confirmed by SDS-PAGE and urea-PAGE, which indicated that DRT2-RT-ncRNA complex had been purified successfully (Supplementary Fig. S1). Utilizing cryo-electron microscopy, we solved the structure of the DRT2 system at a resolution of 3.49 Å. The 2D class averages showed particles as monomers, and the 3D volume was also reconstructed as a monomer, consistent with our purification experiment (Fig. 1C and Supplementary Fig. S2). Owing to the high-quality density map, we were able to trace the complete structure of DRT2 RT protein. The majority of the ncRNA could be resolved, except for the regions 33–123, 150–153, 176–183, and 220–225 nt (Fig. 1D and Supplementary Fig. S3).

The DRT2-RT protein adopts a conventional “right-hand” fold and comprises 14 α-helices and 11 β-strands sequentially numbered from the N-terminus to the C-terminus. The fingers domain contains several α-helices linked by disordered loops and an extended three-stranded antiparallel β-sheet (termed sheet-1). A β-hairpin (β1 and β2) stretches towards the thumb domain but does not make direct contact, creating a cleft for the ncRNA binding (Supplementary Fig. S4A and B). Moreover, α3, α5, and α6 form a hydrophobic pocket into which the α-helix (α9) of palm domain packs (Supplementary Fig. S4C). The palm domain comprises eight β-strands forming sheet-2 and sheet-3, together bracket the detached α-helix (α4) of fingers domain. The α4 is docked into the groove formed by the two sheets, establishing hydrophobic interaction networks (Supplementary Fig. S4D). Furthermore, the thumb domain consists of four helices, which are arranged in parallel, forming a tight helix bundle through extensive hydrophobic contacts (Supplementary Fig. S4E).

The ncRNA, harboring five stem-loops and a pseudoknot, extensively interacts with the thumb and fingers domains of the RT protein (Supplementary Fig. S5A). Sequence alignment of closely related DRT2 ncRNAs revealed high overall conservation, particularly in complementary regions that form base-paired structures, underscoring the importance of these rigid structures in maintaining functional integrity (Supplementary Fig. S5C). Notably, the pseudoknot of the ncRNA stabilizes the overall architecture by forming five Watson-Crick base pairs and noncanonical interactions between the two short RNA segments, G155-C159 and G250-C254, effectively bridging distal regions of the ncRNA sequence from different stem-loops (Supplementary Fig. S5B). Most parts of ncRNA were visible in the cryo-EM density map. In contrast, the stem-loop 2 was almost invisible in the density map, and 2D class averages showed that particles feature a diffuse trailing, possibly representing the unbuilt region, suggesting its inherent flexibility or potential distinct functions (Supplementary Figs S2 and S5A). B-factor analysis revealed that the whole structure exhibits stable protein regions, while ncRNA component exhibits elevated flexibility, suggesting they may exist in a mixed conformational state (Fig. 1E). Thus, we employed 3D classification to isolate discrete states, and we observed that the RT proteins were still identical and the variability only occurred in the ncRNA’s stem-loop 2 regions. In these volumes, the stem-loop 2 exhibited diverse conformations: some formed two opposing extended stem-loops in the stem-loop 2 regions, some displayed these loops at various angles to each other, while others almost lacked these densities (Fig. 1F). These incomplete and poor density maps made it challenging to build a reliable model, confirming the flexibility of this region again. This flexible region likely serves a functional role, potentially acting as the template for reverse transcription, which is consistent with a recent study of the DRT2 system [28].

### DRT2-RT and ncRNA form extensive interactions

**Figure 2.**
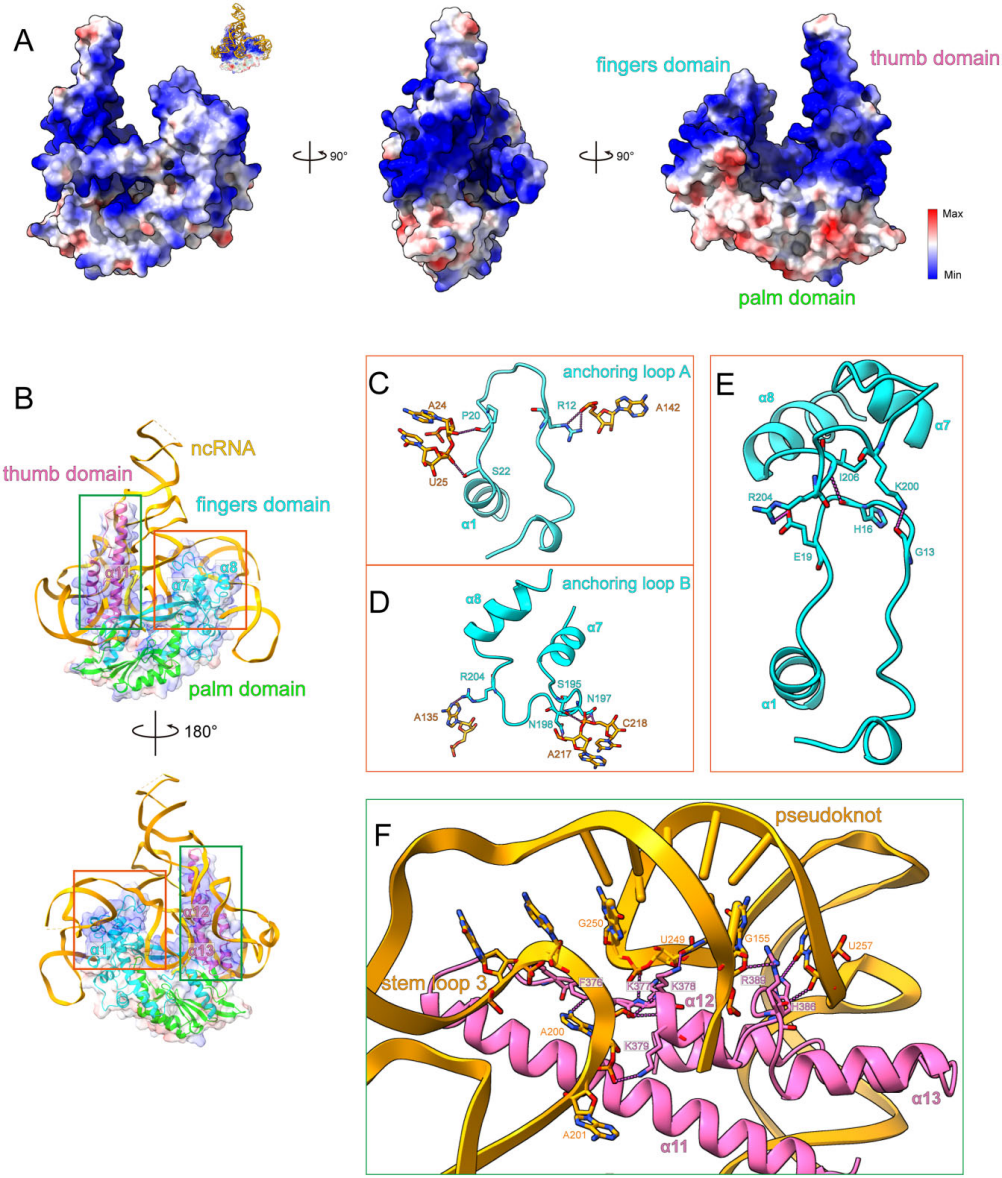
Surface potential and interaction analysis of DRT2 system. (**A**) Electrostatic surface representations of DRT2 system. The abbreviated image of ncRNA binding to RT’s electrostatic surface is shown in the top-right inset. (**B**) Overall structure of DRT2 system. Two distinct regions are highlighted by colored boxes. (**C-E**) Detailed insights into the interactions between anchoring loop A/B and ncRNA and the two loops’ contacts. Key interacting residues are shown in stick representation. The color scheme of the boxes is the same as Fig. 2B. (**F**) Detailed insights into the interaction region surrounding the pseudoknot of ncRNA. Key interacting residues are shown in stick representation.

We analyzed the surface electrostatic potential of DRT2-RT protein and conducted a detailed examination of the interactions between RT and ncRNA. The majority of the protein surface was positively charged, particularly in the thumb and fingers domains, where the ncRNA bound and formed extensive electrostatic interactions (Fig. 2A). Then we identified two distinct regions with notable features from thumb and fingers domains, respectively, were likely to be critical for the assembly of the DRT2 system (Fig. 2B).

The first featured region comprises two intervening loops: one located between the N-terminal and α1, and the other between α7 and α8. The first loop forms a hairpin-like structure that penetrates the ncRNA and establishes polar interactions with the ncRNA. Specifically, R12 appears to establish contacts with U142 in the stem-loop 3, whereas P20 and S22 contact A24 and U25 within stem-loop 2. Through these interactions, the loop anchors the two regions of ncRNA, enhancing the overall structural stability of the ncRNA, thus named anchoring loop A (Fig. 2C). Similarly, the other loop, anchoring loop B, connects stem-loop 2 and stem-loop 4 regions by forming extensive electrostatic interactions. In detail, R204 forms hydrogen bonds with A135 in stem-loop 2. In stem-loop 4, S195 and N197 interact with C218, while N198 contacts A217 (Fig. 2D). More importantly, the two anchoring loops interact with each other via K200-G13, I206-H16 and R204-E19 (Fig. 2E), and together with their interactions with ncRNA, they stabilize the assembly and conformation of the DRT2-RT-RNA complex.

Moreover, ncRNA wraps around the pseudoknot within the thumb domain, forming extensive interactions (Fig. 2B). The linker loop between α11 and α12, along with the initial segment of α12, is lysine-rich, and interacts with the ncRNA’s pseudoknot and stem-loop 3. Specifically, the lysine array containing K377-K379 forms dual interaction networks. On one hand, F376 and K379 appear to form hydrogen bonds with A200 and A201 in stem-loop 3. On the other hand, K377 and K378 interact with U249 and G250 in pseudoknot region. These interactions stabilize the positions and conformations of the two regions of ncRNA, and reinforce the formation of pseudoknot. The intervening loop between α12 and α13 inserts the terminal of pseudoknot and interacts with both chains by forming R389-G155 and H386-U257 interactions (Fig. 2F). Together with the lysine array, the inserting loop sandwiches the pseudoknot through extensive acid-base interactions, facilitating and reinforcing the assembly of the pseudoknot. Collectively, RT’s specialized structures form extensive interaction networks with ncRNA, which stabilize the assembly and provide the structural foundation necessary for functional activity.

### DRT2-mediated catalytic mechanism of RT function

**Figure 3.**
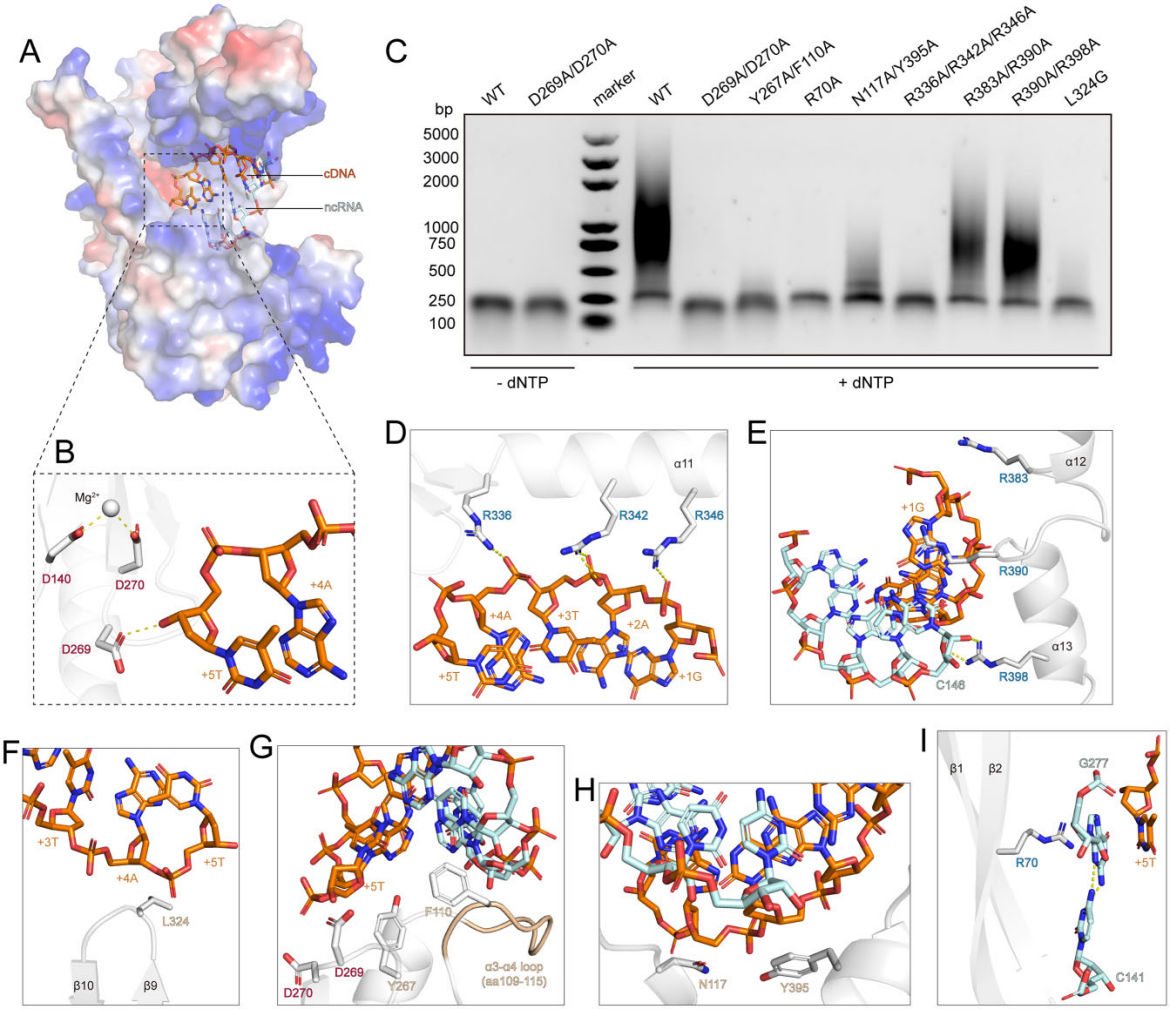
DRT2-mediated RT mechanism. (**A**) Electrostatic surface potential showing the DNA primer-ncRNA duplex bound within a positively charged pocket. (**B**) Close-up view of the catalytic sites. Key residues are shown in sticks. (**C**) RT activity of WT and mutant DRT2 variants. The gel includes a DNA molecular weight marker and represents three independent repeat experiments. (**D-I**) Detailed interactions between DRT2 RT and cDNA-ncRNA duplex. The ncRNA is colored palecyan, and the cDNA is colored orange. Key interacting residues are shown in stick representation.

Next, we tested DRT2 system to validate its functions. After incubating for 30 min with dNTP, DRT2 produced obvious laddered DNA species, confirming that DRT2 possesses reverse transcription activity (Supplementary Fig. S6). Besides, increasing dNTP concentrations led to the accumulation of laddered DNA products in greater amounts and lengths, yielding 0.5-3 kb fragments at 500 μM dNTP. Furthermore, we observed clear density corresponding to several nucleotides, which is linked to the 3′ end of the ncRNA, near the catalytic pocket, likely representing the DNA fragment termed DNA primer [28] (Supplementary Fig. S3E). The nucleotide at the 3′ end of the primer DNA is positioned near the active site (D269), enabling templated DNA synthesis upon dNTP incorporation. Additionally, the density corresponding to a divalent metal ion (Mg²⁺) is observed around the other active site (D270) (Supplementary Fig. S3F). D270 and D140 coordinate with Mg²⁺ and stabilize its position. D269 forms a polar interaction with the nucleotide at 3′ end of DNA primer, reinforcing its conformation for following reverse transcription process. D140, D269, and D270 constitute a DDD catalytic triad, which is a hallmark of RTs’ identification in early research [1, 29–31] (Fig. 3B). Structural superposition and sequences alignment of several representative RTs further demonstrated that RTs share a similar catalytic site featuring two continuous aspartic residues (Supplementary Fig. S7B and C). Mutations of the catalytic residues completely abolished the reverse transcription function, demonstrating that these active sites are indispensable for the enzymatic activity (Fig. 3C). Altogether, these results partially revealed the conserved enzymatic mechanism of RT family.

The DNA primer consists of five nucleotides (5′-GATAT-3′), which form base pairs with nucleotides A142-C146 of the ncRNA, forming a cDNA-ncRNA heteroduplex. The electrostatic potential surface revealed that the duplex is nested within a positively charged pocket (Fig. 3A). The docked duplex is further sandwiched between the thumb domain and the fingers domain, stabilized via extensive interactions. Specifically, R346, R342, and R336, positioned consecutively along α11, form a linear array of contacts with the cDNA backbone at nucleotides 2A, 3T, and 4A, providing extensive electrostatic stabilization (Fig. 3D). Alanine substitutions of these residues (R336A/R342A/R346A) abolished reverse transcription function, suggesting their essential role in stabilizing the duplex (Fig. 3C). At the initial region of the duplex, R383, R390, and R398 are aligned in a row extending toward the duplex, with R398 forming a polar contact with C146 (Fig. 3E). The double mutants R383A/R390A and R390A/R398A showed minimal effects on reverse transcription activity (Fig. 3C), likely because these potential interaction regions are positioned away from the catalytic center, contributing only peripheral stabilization.

Apart from electrostatic interactions, other contacts further reinforce the duplex conformation. L324, arising from the β9-β10 loop, reaches beneath the 3′end of the cDNA, providing structural support (Fig. 3F). Accordingly, the L324G mutant almost abrogated reverse transcription activity (Fig. 3C). The distal portion of the α3-α4 loop (residues 109-115) extends toward the cDNA, forming a platform that supports the duplex. Importantly, F110 inserts into the duplex and stacks against neighboring bases, anchoring the duplex, while Y267, adjacent to the catalytic D269, packs against cDNA nucleotide 5T and likely facilitates cDNA synthesis (Fig. 3G). Consistently, the F110A/Y267A mutant failed to generate cDNA (Fig. 3C). Similarly, the duplex clamps N117 and Y395, which provide structural support and stabilize the overall architecture (Fig. 3H). As anticipated, the reverse transcription activity was greatly compromised by the N117A/Y395A mutant (Fig. 3C). Finally, R70, which protrudes from the β1-β2 hairpin, stacks directly against G277 that occupies the incoming nucleotide-binding site of the catalytic center and forms a noncanonical base pair with C141 [28], likely stabilizing the conformation in the dNTP-free state (Fig. 3I). Replacement of R70 with alanine completely abrogated cDNA synthesis, demonstrating the critical role of the arginine residue in reverse transcription (Fig. 3C).

Together, these findings underscore that the stabilization of the cDNA-ncRNA duplex, reinforced by key residues, is fundamental for DRT2 reverse transcription activity.

### Structural comparison reveals the diversity of RT protein family adaptation

**Figure 4.**
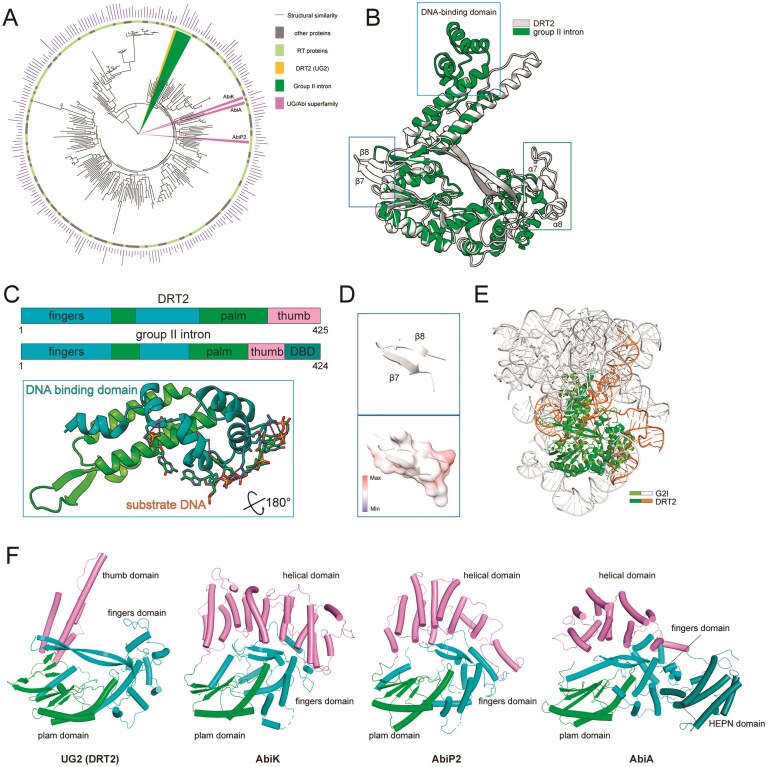
Structural comparisons uncover diverse adaptation patterns in RT protein families. (**A**) Phylogenetic reconstruction is performed using all sequences derived from structurally homologous proteins identified by DALI server. (**B**) Structural comparison of DRT2 and G2I (PDB: 7UIN). Several notable distinctions are highlighted in colored boxes. (**C**) Comparison of the domain architectures of DRT2 and G2I. Close-up view of DNA binding domain of G2I is below. (**D**) Electrostatic surface representation of β7–β8 in DRT2-RT protein. (**E**) Alignment of RNA components of DRT2 and G2I (PDB: 7UIN). (**F**) Structural comparison of DRT2 with UG/Abi family members, including AbiK (PDB: 7Z0Z), AbiP2 (PDB: 7R08), and AbiA (PDB: 8OZ7).

Structural homology search using the DALI server found a total of 2138 homologous structures, with the highest Z-score reaching ∼21.5 [32]. Phylogenetic analysis of all homologous structures identified many members of the RT protein family, with DRT2 forming a distinct evolutionary branch (Fig. 4A). Multiple sequence alignment of selected representative RT proteins with high Z-scores showed the catalytic site of the RT protein superfamily is relatively conserved (Supplementary Fig. S7A). Importantly, based on sequence similarity, the clade containing G2I is most closely related to DRT2, and the several top matches in terms of structural similarity are G2I as well, implying similar characteristics with DRT2. G2I, the most abundant RT type in prokaryotes, is composed of a catalytic ribozyme and an intron-encoded RT, and possesses both RT activity and mobility [33, 34]. Alignment of G2I with our experimentally determined structure demonstrated that the two structures share a conserved “right-hand” configuration but differ in thumb domain (Fig. 4B). Notably, the thumb domain of DRT2 RT adopts a simplified and straightened conformation, likely due to the structural requirement of ncRNA, contrasting G2I RT, which recognizes its substrate DNA for retrotransposition via an additional DNA-binding domain [35] (Fig. 4C). Furthermore, the fingers domain of DRT2 has acquired specialized functional elements differing from G2I, such as α7, α8, and their linker loop, to mediate interactions with ncRNA (Fig. 2B-E). Additionally, the palm domain of DRT2 features several β-sheets (β7-β8), which undergo the disorder-to-order transition compared to G2I, suggesting a potential functional divergence. The surface electrostatic potential showed that these β-sheets are electroneutral or even electronegative, implying this region serves as additional functional roles (Fig. 4D). Besides, structural superposition of the RNA components of the two systems exhibits substantial differences (Fig. 4E). G2I’s RNA is large and highly structured, consisting of six domains (D1-D6), with D5 functioning as the catalytic core for splicing and retrotransposition [36]. DRT2 ncRNA is compact, folding into five stem loops that serve as the scaffold for RT-ncRNA complex stability and the template for following reverse transcription [28, 37]. These distinct structures indicate that DRT2 is repurposed by prokaryotes for specialized functions, while losing its mobility during evolution. Together with the closer phylogenetic relationship, these structural findings imply that DRT2 may have evolved from G2I. The functional transition of DRT2 from G2I’s ancestral role in mobility to its specialized function parallels other well-documented cases of RT adaptation, such as the evolution of telomerase from non-LTR retrotransposons [2, 38–40].

Moreover, phylogenetic analysis showed that DRT2 shares moderate homology with several canonical UG/Abi RTs, including AbiA, AbiK, and AbiP2 (Fig. 4A). And structural alignments yielded RMSDs of 4.54, 4.53, and 7.74 Å, suggesting conservation across the palm and fingers domains. Contrastingly, their thumb domains are notably different. DRT2 adopts a straight, extended thumb domain that provides a scaffold for ncRNA binding, whereas UG/Abi RTs feature helical or HEPN domains that are rich in parallel-arranged helices (Fig. 4F). These helical or HEPN domains allow UG/Abi RTs to assemble into higher-order structures, including AbiK hexamers, AbiP2 trimers, and AbiA dimers through protein-protein contacts [41, 42]. Correspondingly, the structural difference indicated functional divergence. DRT2 functions as a monomer and relies on ncRNA to mediate reverse transcription, in contrast, UG/Abi RTs can synthesize DNA without additional nucleic acid cofactors. The ncRNA dependence of DRT2 likely represents a more precise defense mechanism.

Collectively, RTs have maintained conserved catalytic cores throughout evolution while acquiring domain-specific adaptations that enable functional diversification, revealing the evolutionary plasticity of the RT protein family.

## Discussion

The widespread adaptation of RT proteins across prokaryotes highlights their remarkable evolutionary plasticity. Our structural and functional characterization of the DRT2 system provides compelling evidence for how this RT-ncRNA binary system has been specialized for prokaryotic defense mechanisms while maintaining the core catalytic characteristic of RT proteins. In our study, we elucidate the structural basis of DRT2 system at a resolution of 3.49 Å and reveal how its RT domain engages with a structured ncRNA component through extensive interactions. *In vitro* reverse transcription assay showed that the catalytic mechanism of cDNA synthesis is mediated by conserved catalytic residues. Additional mutagenesis studies demonstrated that the stability of the DNA primer-ncRNA duplex is critical for reverse transcription. Phylogenetic and structural analyses highlight the evolutionary diversity of the RT protein family. Besides, DRT2’s adaptations, like a straight extended thumb domain and several distinct structures, develop the function to contact ncRNA that couples the reverse transcription to phage defense, while preserving ancestral RNA-binding capacity.

Under physiological conditions, the cDNA primer is covalently attached to the 3′ end of the ncRNA. This linkage constrains reverse transcription to begin at a fixed site, coupling ncRNA structure with the precise onset of cDNA synthesis. By physically tethering the primer to the RNA template, DRT2 system avoids randomness of de novo priming and ensures fidelity during reverse transcription. Such an arrangement likely reduces the risk of aberrant cDNA products, accelerates the onset of reverse transcription-mediated antiphage activity, and enhances the efficiency of defense. Future studies should further address the impact of primer tethering on RT dynamics and the potential functions of the cDNA beyond antiphage defense.

The research on DRT2’s ncRNA-dependent mechanism has offered new insights into strategies of prokaryotic immunity. Unlike previously reported defense-associated RTs, DRT2’s reliance on cognate ncRNA suggests a new regulatory mechanism in prokaryotic defense systems [6, 8]. Although DRT2 and G2I share homologous architectures and domain distributions, the ncRNA drives the evolution of specialized structures, emphasizing the important role of ncRNA in defense functions. Thus, further exploration of similar defense systems remains necessary to broaden our understanding.

During the preparation of our manuscript, two studies also characterized the DRT2 system in detail [28, 37]. Compared to our structure, the previous model captures 46 extra nucleotides in stem-loop 2 region, forming one complete stem-loop and one partial stem-loop, additionally, partially consistent with our 3D classification results. However, these extra stem-loops of some classified volumes feature varying orientations, indicating potential functional states (Fig. 1F and Supplementary Fig. S8A). Moreover, alignment of our structure and its dNTP-bound structure shows that the D270 shifts ∼2.5 Å toward the cDNA, causing a series of interaction changes, further elucidating the DRT2-mediated reverse transcription mechanism (Supplementary Fig. S8B).

In summary, our study presents detailed structural and functional investigations of the DRT2 system, providing significant mechanistic and evolutionary advancements and offering new insights into the diversification of prokaryotic antiviral strategies.

## Supplementary Material

gkaf1135_Supplemental_File

## Data Availability

The atomic coordinate has been deposited in the Protein Data Bank under accession codes 9JL3 (DRT2-RT-ncRNA binary complex). Cryo-EM map has been deposited in the Electron Microscopy Data Bank under corresponding accession code EMD-61577.
