# Supplementary material for: Molecular mechanism of the type 2 defense-associated reverse transcriptase
Source: Nucleic Acids Res. 2025 Nov 8;53(20):gkaf1135. doi: 10.1093/nar/gkaf1135 (PMC12596737; doi:10.1093/nar/gkaf1135)
Supplement: gkaf1135_Supplemental_File [file gkaf1135_supplemental_file.pdf]

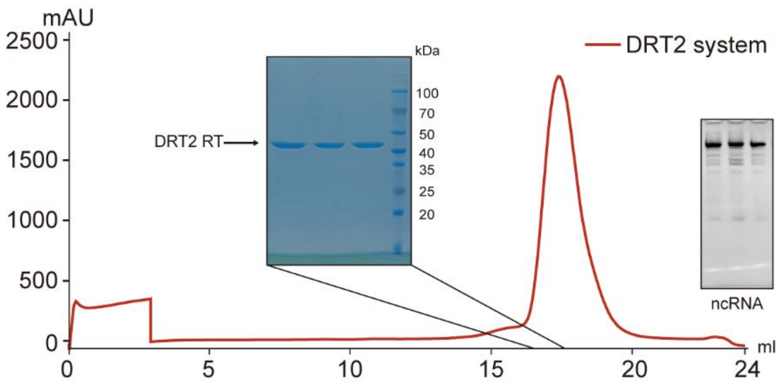

Figure S1. The size exclusion chromatograms of DRT2 system in Superose 6 Increase column.

The peak fractions containing DRT2 complex are analyzed by SDS-PAGE and urea-PAGE. The gels are representative of three repeat experiments.

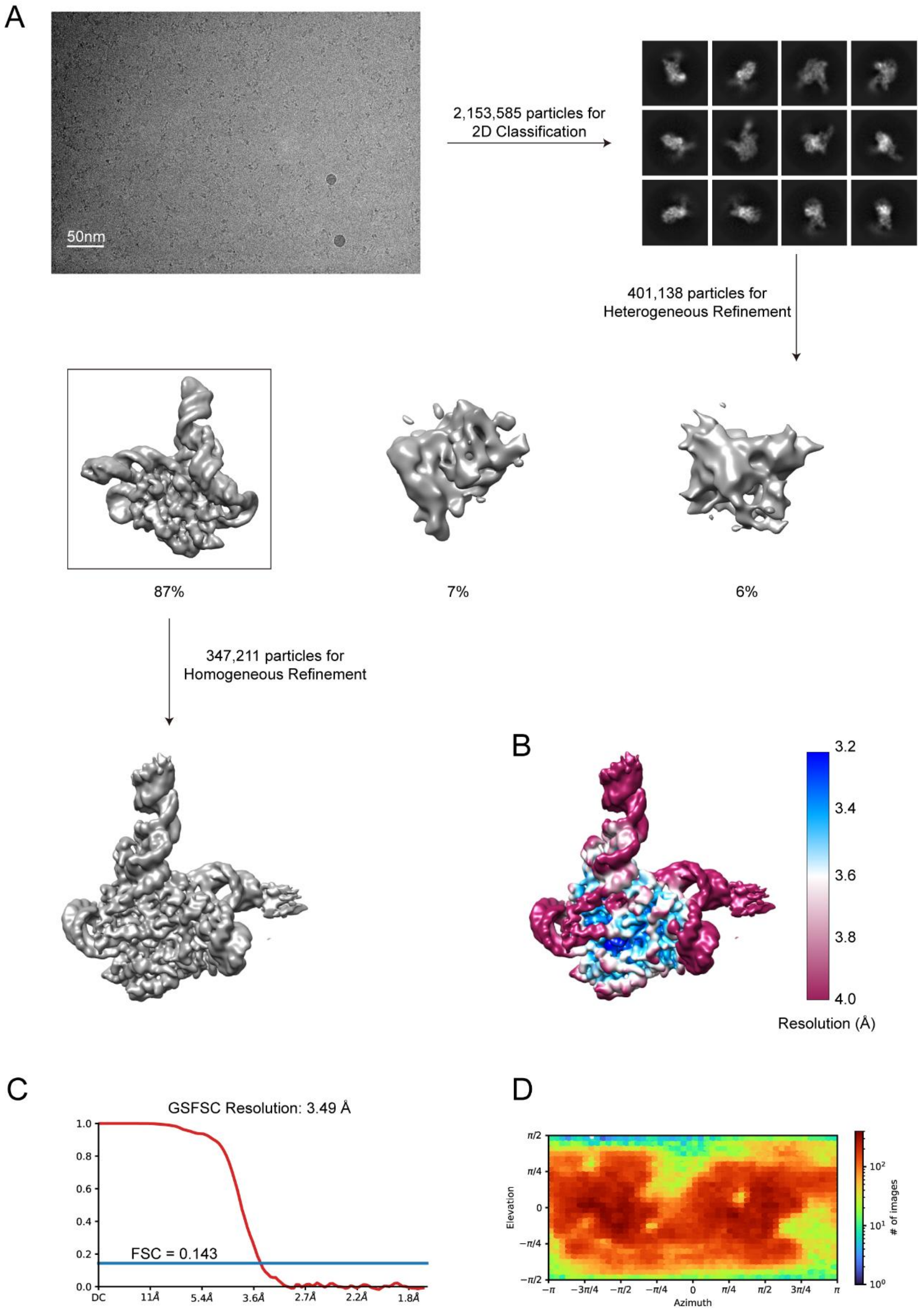

Figure S2. Cryo-EM data processing of DRT2 system.

(A) The flowchart of DRT2 system data processing. (B) Local resolution estimation of the cryo-EM density map, presented alongside its corresponding resolution scale. (C) The Fourier Shell Correlation (FSC) curve of the cryo-EM density map, using the gold-standard cutoff of FSC=0.143. (D) Viewing direction distribution of the particles in the DRT2 system's map.

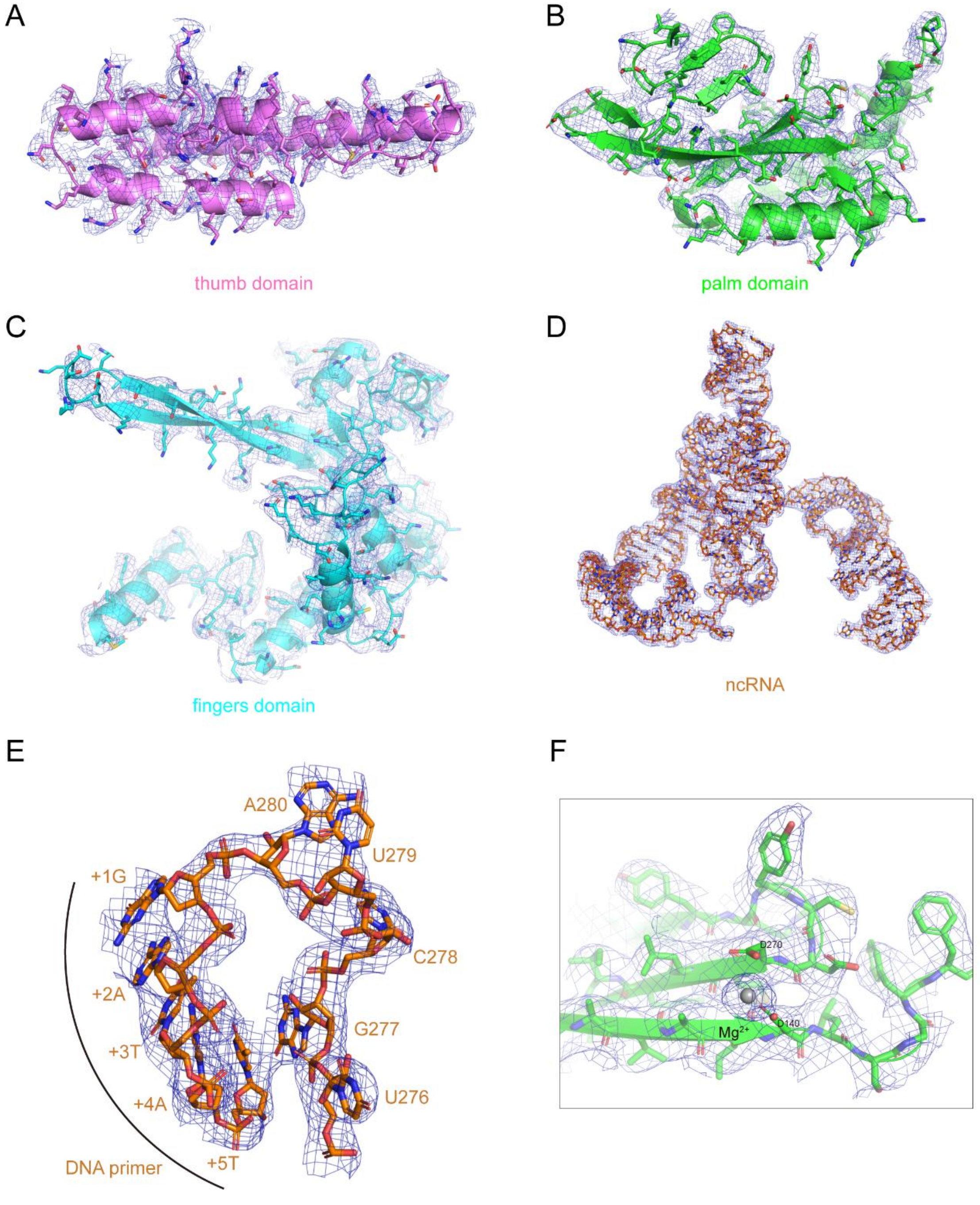

Figure S3. Atomic models of the DRT2 complex components were fitted into the corresponding cryo-EM density map.

(A-C) Cryo-EM density map and models of DRT2 RT protein. (D-E) Cryo-EM density map and models of DRT2 ncRNA and DNA primer. (F) Cryo-EM density of Mg^{2+} and interacting residues.

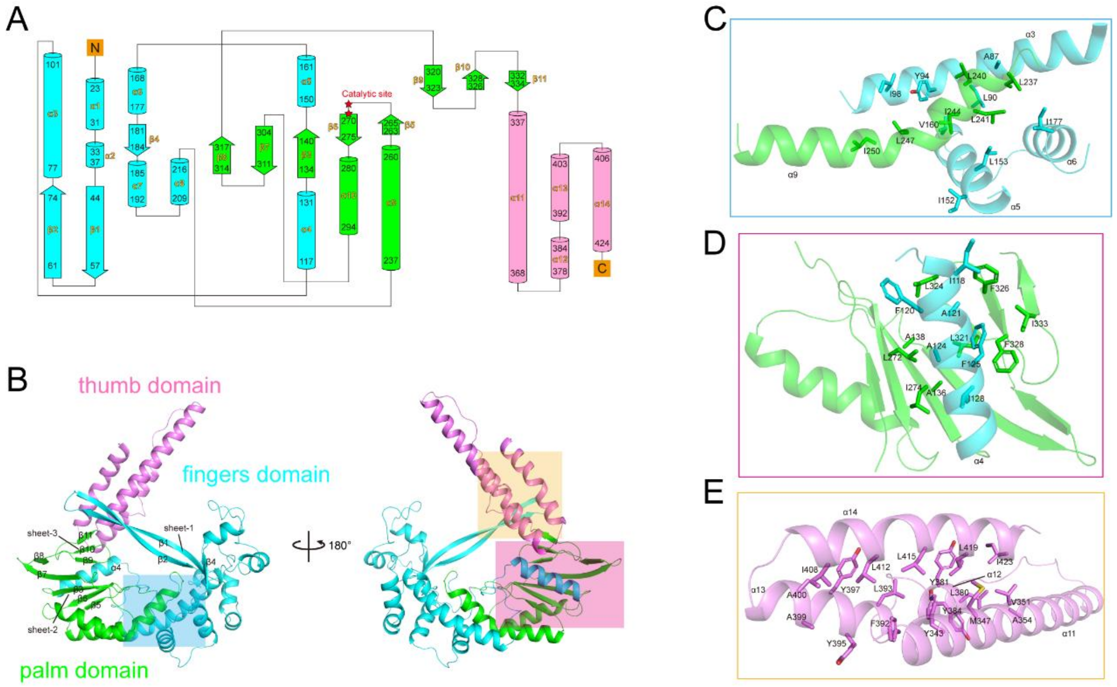

Figure S4. Overall structure of DRT2-RT protein.

(A) Topological structure of DRT9-RT protein. The domain color is consistent with Fig. 1d, and the catalytic residues are highlighted with pentagram symbols. (B) The overall structure of DRT2-RT protein. (C-E) Detailed insights into the interactions within RT protein. Key interacting residues are shown in stick representation.

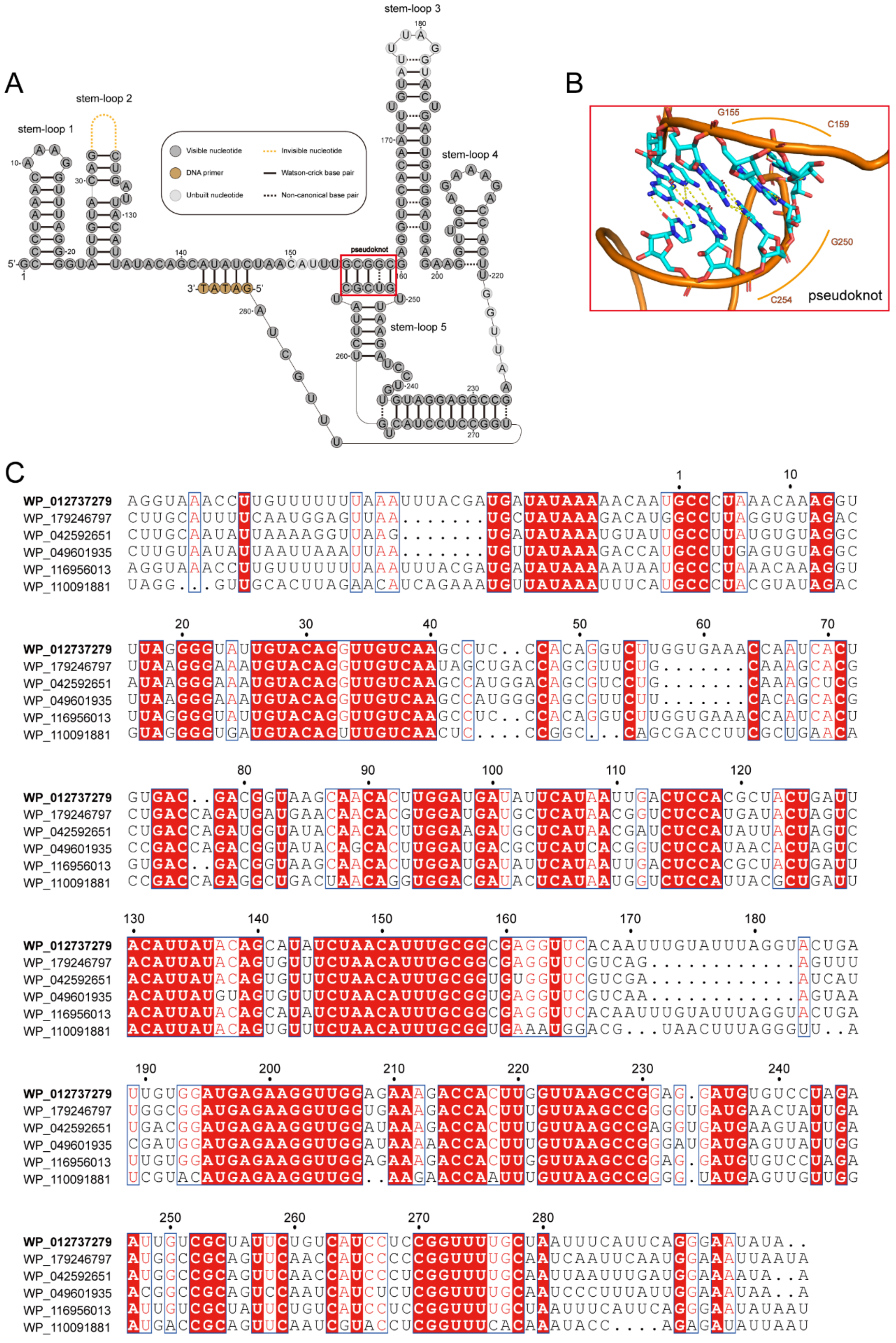

Figure S5. Structural features and conservation of the DRT2 ncRNA.

(A) Secondary structure diagram of the DRT2 ncRNA. (B) Detailed insights into the pseudoknot of ncRNA. (C) DRT2 ncRNA conservation analysis (WP_012737279, this study).

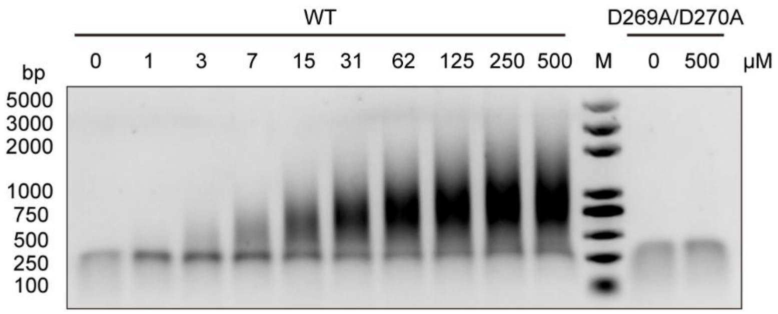

Figure S6. Reverse transcription assay with a dNTP concentration gradient.

The purified complex was incubated with a gradient of dNTP concentrations (0, 1, 3, 7, 15, 31, 62, 125, 250, and 500 µM). The resulting DNA products were visualized using agarose gel electrophoresis, with lane M represents the DNA molecular size marker. The gel is representative of three repeat experiments.

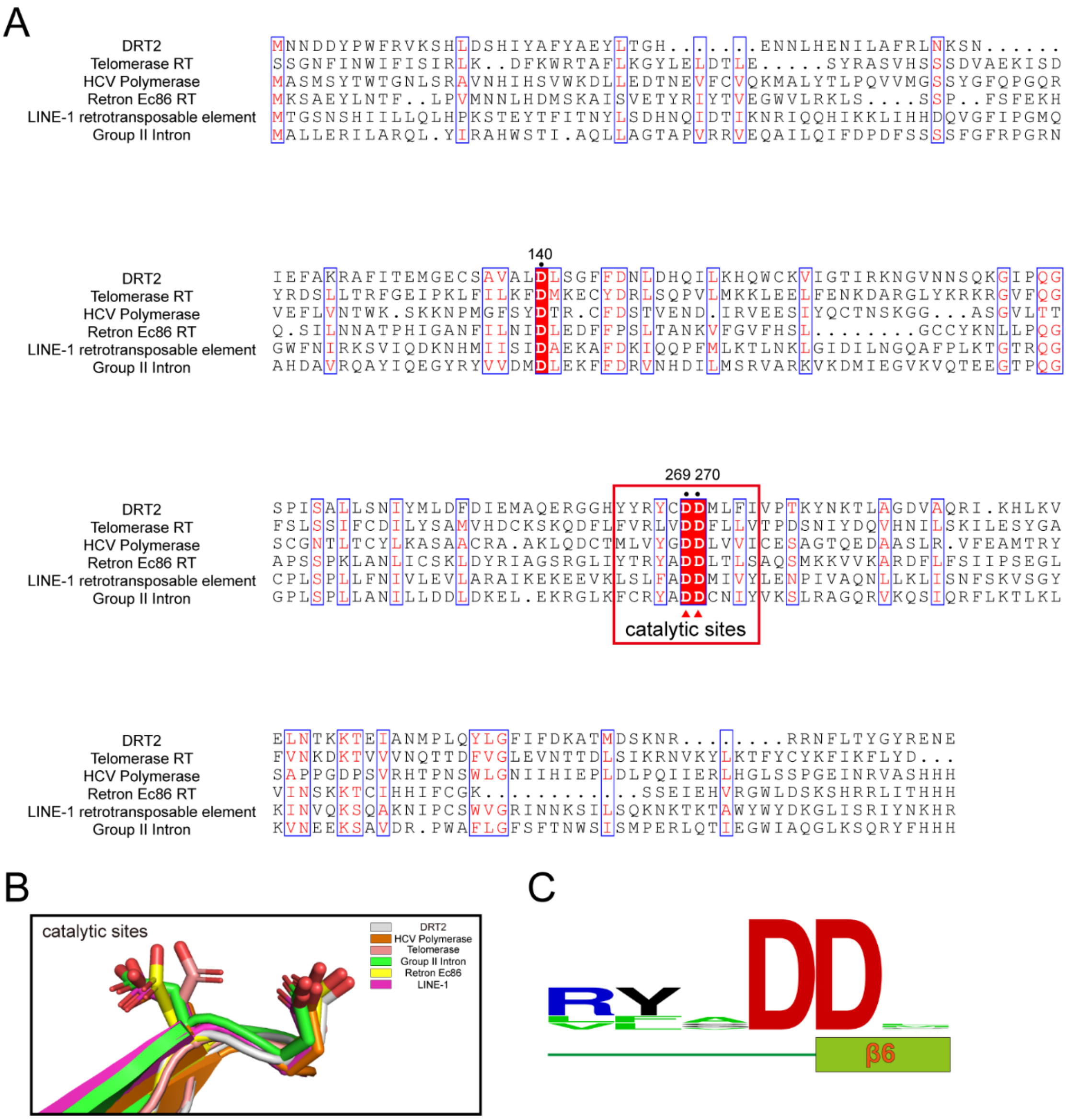

Figure S7. Conservation analysis of catalytic sites in DRT2.

(A) Multiple sequence alignment (MSA) of RT proteins. Representative sequences are derived from structurally homologous proteins identified by DALI server. (B) The alignment of representative RTs' catalytic sites. (C) Sequence logo representing the conserved catalytic sites of representative RTs.

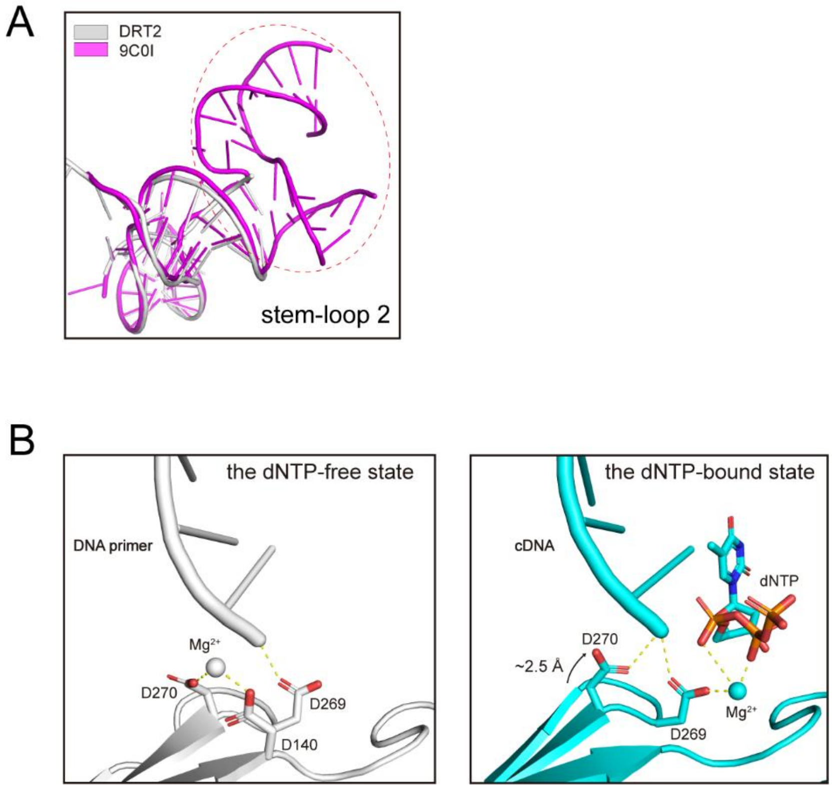

Figure S8. Structural comparisons of DRT2 models.

(A)The alignment of stem-loop 2 between DRT2 and a previously reported model (PDB: 9C0I).

(B) The comparison of the structures of DRT2 and its dNTP-bound state (PDB: 9C0J). Key interacting residues are shown in stick representation.

Table S1. Cryo-EM data collection, refinement and validation statistics

	DRT2-RT-ncRNA binary complex<br>(EMDB-61577)<br>(PDB 9JL3)
**Data collection and processing**	
Magnification	105,000
Voltage (kV)	300
Electron exposure ($e^{-}/Å^2$)	54
Defocus range (-µm)	1.2 - 2.2
Pixel size (Å)	0.85
Symmetry imposed	*C1*
Initial particle images (no.)	2,153,585
Final particle images (no.)	347,211
Map resolution (Å)	3.49
FSC threshold	0.143
Map resolution range (Å)	3.2 - 4.0
**Refinement**	
Initial model used	AlphaFold
Model resolution (Å)	3.5
FSC threshold	0.5
Model resolution range (Å)	3.4 - 4.3
Map sharpening B factor ($Å^2$)	-155.4
Model composition	
Non-hydrogen atoms	7320
Protein residues	425
Nucleotides	179
B factors ($Å^2$)	
Protein	94.81
Nucleotide	79.49
R.m.s. deviations	
Bond lengths (Å)	0.013
Bond angles (°)	1.687
Validation	
MolProbity score	1.64
Clashscore	9.19
Poor rotamers (%)	0.52
Ramachandran plot	
Favored (%)	97.16
Allowed (%)	2.84
Disallowed (%)	0.00
